# The level of I-FABP and IgA/IgG to beta-lactoglobulin in mothers at risk for gestational diabetes and in their children: association with antibodies to *Bifidobacterium adolescentis* and *Bifidobacterium breve*

**DOI:** 10.3389/fimmu.2025.1613002

**Published:** 2025-07-02

**Authors:** Tamara Vorobjova, Aili Tagoma, Celeste Peterson, Ija Talja, Anu Bärenson, Kristi Alnek, Helis Janson, Kaja Metsküla, Anne Kirss, Epp Sepp, Tiiu Rööp, Siiri Kõljalg, Raivo Uibo

**Affiliations:** ^1^ Department of Immunology, Institute of Biomedicine and Translational Medicine, University of Tartu, Tartu, Estonia; ^2^ Department of Pediatrics, University of Tartu, Children’s Clinic of Tartu University Hospital, Tartu, Estonia; ^3^ Women’s Clinic, Tartu University Hospital, Tartu, Estonia; ^4^ Department of Microbiology, Institute of Biomedicine and Translational Medicine, University of Tartu, Tartu, Estonia

**Keywords:** gestational diabetes mellitus, I-FABP, IgA and IgG to beta-lactoglobulin, IgA and IgG to *Bifidobacterium*, ELISA, immunoblot, flow cytometry

## Abstract

**Background:**

The mechanisms underlying gestational diabetes mellitus (GDM) and their impact on maternal and child immunity remain unclear. We hypothesize that gut microbiome alterations and increased small intestinal permeability contribute to GDM. Intestinal fatty acid-binding protein (I-FABP) leakage and elevated IgA/IgG against beta-lactoglobulin may indicate mucosal damage and may serve as biomarkers.

**Methods:**

This study evaluated I-FABP and IgA/IgG levels against beta-lactoglobulin in mothers with and without GDM (n=100) and in their children (n=87 at time point 1 (TP1), n=79 at time point 2 (TP2). Levels of antibody to *Bifidobacterium adolescentis* (DSM20083, DSM20086) and *Bifidobacterium breve* (DSM20213) were assessed using flow cytometry. I-FABP was measured using the Hycult Biotech ELISA Kit, and IgA/IgG levels to beta-lactoglobulin were measured using in-house ELISA.

**Results:**

I-FABP and IgA/IgG levels did not significantly differ between mothers with and without GDM. However, children at TP1 had significantly higher I-FABP, IgA and IgG levels to beta-lactoglobulin than their mothers (p<0.01). In children, both I-FABP and IgA levels to beta-lactoglobulin declined with age (p<0.05). The children of mothers with GDM had higher IgA levels to beta-lactoglobulin (p=0.004). I-FABP was inversely correlated with IgA to *B. adolescentis* in GDM mothers (p<0.002). Breastfeed children had higher beta-lactoglobulin IgA/IgG levels (p=0.02), but I-FABP levels did not differ regarding the length of breastfeeding.

**Conclusion:**

Higher I-FABP and IgA levels to beta-lactoglobulin in children suggest increased intestinal permeability compared to adults. Only IgA to beta-lactoglobulin was significantly elevated in the children of mothers with GDM.

## 1 Introduction

Gestational diabetes mellitus (GDM) is a glucose intolerance detected initially during pregnancy (1). The prevalence of GDM in Europe varies from 2 to 6% (1, 2) while there has been a rapid global increase, with a pooled standardized worldwide prevalence estimated at 14.0% (3). In Estonia, GDM was diagnosed in 6% of pregnant women based on glucose tolerance testing (GTT) at gestational weeks 24–28 (4).

Intestinal fatty acid-binding protein (I-FABP) is an intracellular protein that is specifically found in the epithelial cells of both the small and large intestines (5, 6). When I-FABP leaks from the mature villous epithelium into the bloodstream, it may serve as a marker for intestinal mucosal injury and compromised gut barrier function (5, 6). Increased intestinal permeability has been proposed as a key factor in the development of type 1 diabetes (T1D) (7). Elevated level of I-FABP was shown as a serological marker indicating epithelial damage in pediatric T1D (8).

In our previous study (9), which examined the level of fatty acid-binding protein 4 (FABP4) and the level of intestinal fatty acid-binding protein (I-FABP) in pregnant women with GDM and in pregnant women belonging to the GDM risk group as controls, we showed that FABP4 and I-FABP levels were not dependent on the diagnosis of GDM, but rather on body mass index (BMI).

Elevated levels of antibodies to cow’s milk components, including antibodies to bovin beta-lactoglobulin, casein and bovin serum albumin, have been reported in patients with T1D (10–14), leading to increased gut permeability and its possible association with the pathogenesis of T1D (7, 14, 15). Moreover, the complex interplay between gut permeability, mucosal immune response and intestinal microbiota has been shown in the pathogenesis of T1D (7, 16, 17).

The role of bifidobacteria in enhancing intestinal barrier function through the stabilization of claudins and occludins at tight junctions, as well as in maintaining the integrity of intestinal microvilli, has been demonstrated in both mouse models and humans (18–20) The importance of bifidobacteria in the development of beta-cell autoimmunity in children was shown in a study of de Goffau et al., 2013 (21).

There is ample evidence about the role of gut microbiota alterations in the pathogenesis of GDM (22, 23) demonstrating that the precursors of GDM originate in the gut microbiota and GDM is yielded through microbiota-induced inflammation. Importantly, it has been shown that the maternal GDM status can influence the composition of the neonatal gut microbiota (24, 25).

The aim of present study was to evaluate the level of I-FABP and the level of IgA and IgG to beta-lactoglobulin in mothers and their children in the Gestational Diabetes Mellitus (GDM) pregnancy cohort recruited from the Woman’s Clinic of Tartu University Hospital taking in consideration their BMI, level of IgA/IgG to *B. adolescentis* (strains DSM20083 and DSM20086) and *B. breve (*strain DSM20213). Furthermore, we aimed to determine whether I-FABP and IgA/IgG antibodies’ level was associated with children’s age, diagnosis of atopic dermatitis or asthma and length of breastfeeding, as well as the month of introduction of cow’s milk.

## 2 Materials and methods

### 2.1 Subject recruitment and sample collection

Within the HEDIMED (Human Exposomic Determinants of Immune Mediated Diseases) study the mothers of the Gestational Diabetes Study group (GDS) and their children were recruited from the Women’s Clinic of Tartu University Hospital between September 2020 and August 2021. All participants belonged to the GDM (Gestational Diabetes Mellitus) risk group during pregnancy according to the Estonian Gynaecologists’ Society guidelines. The criteria for the GDM risk group were as follows: pre-pregnancy obesity (body mass index ≥ 30 kg/m^2^); history of GDM; history of impaired glucose tolerance; first-degree relative with diabetes; previous delivery of a macrosomia (>4500 g); history of unexplained fetal death; and history of polycystic ovary syndrome (4).

All women performed a 2-hour 75g oral glucose tolerance test between 24 and 28 gestational weeks (in one case at 29 weeks of gestation) and GDM was diagnosed if at least one of the following criteria was fulfilled: fasting plasma glucose ≥ 5.1 mmol/l; 1-h plasma glucose ≥ 10.0 mmol/l; 2-h plasma glucose ≥ 8.5–11.0 mmol/l.

Women who had previously been diagnosed with diabetes, whether Type 1 or Type 2, were excluded from the study.

A total of one hundred mothers and their children were investigated. Forty- four mothers diagnosed with GDM during pregnancy comprised the GDM group (median age 34.75) along with their children, while 56 mothers who were not diagnosed with GDM were classified, together with their children, as the non-GDM group (median age 34.25). The mothers and their children (median age 1.95 (1.30-4.92) were invited to visit the study pediatrician twice, with one year between each visit. During the appointments, blood samples were collected from both the mothers and their children. At the first visit (time point 1: TP1) only 87 blood samples were obtained from the children recruited to this project (**Table 1**).

**Table 1:** The number and median age of the mothers and their children in the groups studied for the level of I-FABP and/or IgA/IgG to beta-lactoglobulin.

Groups studied	n	Median age (IQR 25%-75%)	Mann-Whitney p
Mothers with previous history of GDM (GDM)	44	34.75 (32.27-37.38)	
Mothers without GDM (non- GDM)	56	34.25 (31.58-38.67)	0.97*
Their children at time point 1 (TP1)	87	1.95 (1.30-4.92)	
male	49	2.0 (1.20-5.40)	
female	38	1.91 (1.33-2.73)	0.54
Their children at TP2 (TP2)	79	2.95 (2.32-3.87)	
male	41	2.97 (2.37-4.02)	
female	38	2.87 (2.30-3.73)	0.55

The Mann-Whitney p values for the studied groups.

This study was approved by the Ethics Review Committee on Human Research of the University of Tartu (229/M‐16, 23.09.2013, 254/M‐16, 21.12.2015, 298/M-21, 18.11.2019 and 315/M18, 15.05.2020) and was conducted in accordance with the Declaration of Helsinki. Written informed consent was obtained from each participant and from the children’s parents before participation in the study.

### 2.2 Evaluation of serum intestinal fatty acid-binding protein

I-FABP levels were evaluated in the sera of forty mothers with previous history of GDM (n=19; median age 35.08 (interquartile range IQR 31.67-36.67)) and in mothers without previous history of GDM (non-GDM) (n=21, median age 32.83 (IQR 29.63-38.71)] as well as in the sera of their children at time point 1 (TP1) n=87 (median age 1.95 (1.30-4.92) and at time point 2 (TP2) n=79 (median age 2.95 (2.32-3.87).

I-FABP level was evaluated by using the Hycult Biotech HK406–02 ELISA Kit. Serum samples were diluted at 1:10. The results were expressed in pg/ml and were calculated according to the manufacturer’s instructions (the concentration read from the standard curve multiplied by the dilution factor 10).

### 2.3 Evaluation of anti-beta-lactoglobulin IgA and IgG

The IgA and IgG antibodies to beta-lactoglobulin were evaluated in the sera of 100 mothers and in their children at TP1 (n=86).

Serum IgG and IgA antibodies to bovine-lactoglobulin (Sigma-Aldrich) were measured using in- house ELISA test according to the protocol of Savilahti et al., 1993 (11) with modifications. The wells of microtiter plates (Nunc PolySorp, Denmark) were coated with bovine-lactoglobulin at a concentration of 2 μg/ml in a carbonate-bicarbonate buffer, and incubated overnight at 4°C. After washing with Phosphate-Buffered Saline (PBS-Tween 20 (0.05%), the plates were blocked with a 1% normal horse serum in PBS for 1 h at 37°C. The sera were diluted to 1: 20 in a 1% horse serum-PBS, applied to the plate in triplicate (two coated wells and 1 well without the antigen) and incubated for 1 h at 37°C. After washing, 100 μl of horseradish peroxidase-conjugated rabbit anti-human IgA (Dako, Glostrup, Denmark; dilution 1: 4000) and rabbit anti-human IgG (Dako; dilution 1: 4000) were applied to the wells, followed by incubation for 1 h at 37°C. After washing, 100 μl of OPD (o-phenylenediamine dihydrochloride) (ThermoFisher) in acitrate buffer and H_2_O_2_ (Sigma-Aldrich) were applied to the plates and incubated for 10 min at room temperature. The reaction was stopped with 1 N H_2_ SO_4_, and optical density was measured immediately at 492 nm. The absorption value in the well without the antigen was subtracted from the absorption values of the antigen-coated wells, which yielded the absorbance value of the studied sera. Four sera positive for IgE to milk protein (f2, Phadia) were used for the reference pool. The results were presented in arbitrary units (AU) calculated as follows: the values of the studied sera subtracted from the blank were divided by the pool values subtracted from the blank.

### 2.4 Evaluation of IgG4 to the beta-lactoglobulin component f77 nBos d5

The presence of the specific IgG4 to beta-lactoglobulin allergen component f77 nBos d 5 (catalog number 14-4289-01, Thermo Fisher Scientific, Uppsala, Sweden) was detected on ImmunoCAP 250 (Phadia Thermo Fisher Scientific, Uppsala, Sweden). Measurement range: 0.3–30 mg_A_/l. Samples showing values higher than those of the calibration curve were measured once more using additional dilution.

### 2.5 Growth of bacteria

*B. adolescentis* strains DSM20083 and DSM20086 and *B. breve* strain DSM20213 were isolated from the newborn’s feces. All strains were grown on FAA (Fastidious Anaerobe Agar) plates, which were prepared by using the LAB090 medium (LAB M Limited, United Kingdom) and sheep blood. Incubation on an agar plate took place at +37°C for 48 h in an anaerobic growth medium (85% N_2_, 10% H_2_, 5% CO_2_). The bacteria were then collected in a sterile PBS solution (sterile Dulbecco’s 1x phosphate-buffered saline without calcium and magnesium, pH 7.4, Corning, USA), suspended and centrifuged at 1500 g, +4°C for 10 min. The precipitate was resuspended in PBS (for immunoblot) or a 20% glycerine solution (for flow cytometry) and stored at -80°C.

### 2.6 Evaluation of antibacterial IgA and IgG antibody reactivity on a flow cytometer

In order to inactivate the complement in the blood serum, the serum was incubated at +56°C for 30 min. The samples were then centrifuged at 16,000 g for 5 min at +4°C to precipitate the antibody complexes in the serum. The supernatant was transferred to a 0.22 µm spin filter (VWR Centrifugal Filters, US) and centrifuged at 14,000 g for 1 min. The inactivated sera were stored at -20°C before analysis.

To detect bacterial surface antibodies, 25 µl of the bacterial solution (5x10^6^ cells per sample) were incubated in a 96-well V-shaped microtiter plate with 25 µl of serum samples. For this, serum dilution at 1:27 was used, as the strongest and most distinct signals for measuring IgA and IgG antibodies were obtained at this dilution. Incubation of the binding of antibodies in the serum to the bacterial surface antigens took place for 1 hour at +4°C or 15 min at room temperature. Unbound antibodies were removed by adding a PBS-BSA buffer to the microtiter plate twice in a row and centrifuged at 3220 g for 10 min +4°C.

Fluorescently labeled secondary anti-isotype antibodies anti-IgA (Fluorochrome FITC, Jackson ImmunoResearch, USA), anti-IgG (Fluorochrome APC, Jackson ImmunoResearch, USA) were then added and the dilutions for antibodies were 1:200 and 1:214, respectively. After 15-minute incubation at room temperature, washing was repeated under similar conditions as previously described. SYTOX orange nucleic acid dye (dilution 1/1000, Invitrogen™, USA) with Triton X-100 (dilution 1:1000, Sigma-Aldrich, USA) was used and incubated at room temperature for 20 min. The samples were analyzed using flow cytometry LSRFortessa (BD Biosciences, USA) and the results were expressed in median fluorescence intensity (MFI) units (26).

### 2.7 Immunoblot analysis of IgA and IgG bands to *B. breve* strain DSM 20213

Bacterial cells (*Bifidobacterium breve* strain DSM 20213) were suspended in PBS and disrupted with 0.1 mm glass beads (Biospec Products, USA). The protein concentration of samples was estimated by using a Protein Assay solution (Bio-Rad, USA). Equal amounts of proteins from different preparations were mixed with 200-300 μl of a SDS-PAGE sample buffer (62.5 mM Tris (pH 6.8), 2.3% SDS, 5% 2-mercaptoethanol, 10% glycerine, few grains of bromphenol blue) and were heated for 15 min at 95°C. For each gel, approximately 100 μg of total proteins were loaded.

The antigens were separated on a 5-20%- gradient gel with a 5% concentrating gel for the immunoblot according to Nilsson et al., 1997 (27). The antigen (approximately 100 μg per gel) was diluted in a sample buffer (0.5 M Tris-HCl [pH 6.8], 0.5% bromphenol blue, 8% glycerol, 4% SDS, 4% 2-mercaptoethanol), and the mixture was heated at 95° C for 15 min. After cooling, the proteins were loaded onto the gel and were separated for 5.5–6 h at 40 mA and voltage 200 V. On the gels, All Blue 10–250 kDa molecular weight markers (Bio-Rad) were used. The vertical electrophoresis system Hoefer SE-600 was used, and electrophoresis was carried out at current 40 mA and voltage 200 V for 5.5–6 h. Separated proteins were transferred onto a polyvinylidene fluoride (PVDF) membrane (0.45 μm pore size Millipore Intertech, Bedford, Mass) using the semi-dry Hoefer electroblotter equipment (Ancos, Vig. Denmark) for 1.5 hours at a current density 1 mA cm^-2^. A Tris/glycine/acetone transfer buffer, including 0.001% sodium dodecyl sulfate (SDS), was used according to Nilsson et al. (1997) (27). The membrane was saturated by incubation twice for 15 min. each time in blocking buffers I and II. After transfer, the saturated membranes were blocked twice: first with a solution containing polyvinyl pyrrolidone (PVP) and methanol in an ethanolamine/glycine buffer (for 10 min), and next with an etanolamine/glycine buffer containing Tween-20 and gelatine hydrolysate (for 10 min). From a membrane, 3–4 mm strips were cut. Serum was diluted IgA 1/50 and IgG 1/100 in an incubating buffer (containing 1.25 g/l gelatin hydrolysate, 0.25 g/l Tween-20, 6.1 g/l NaCl (Merck, USA), 0.06 g/l Tris Base), and was left on a shaker overnight at +4°C. Before adding polyclonal anti-human IgG or IgA rabbit antibodies, labeled with horseradish peroxidase (HRP) (Invitrogen, USA) (diluted 1:2000), the strips were washed three times with the incubating buffer each 5 min. Incubation with the HRP- conjugated anti-human antibodies lasted for two hours at constant shaking at room temperature. Before the development with carbazole in a 50 mM sodium acetate buffer (pH 5.0) in the presence of hydrogen peroxide during 30 min (0.04% 3-amino-9-ethylcarbazole and 0.015% hyrogen peroxide), the strips were washed three times for 5 min each with an incubation buffer. After 30 min, the reaction was stopped by washing the strips with distilled water.

### 2.8 Statistical analysis

The results obtained for the different study groups were presented as medians with the interquartile (IQR) range (25%-75%). Statistical calculations were performed using Graph Pad Prism 5.0 software, employing the nonparametric Mann-Whitney *U* test (for bivariate comparisons of two continuous variables in the case of a non-normal distribution) and Wilcoxon non-parametric rank test for comparison of the median values of I-FABP levels in two children’s samples at two different time points. For correlation analyses, Spearman nonparametric rank correlation analysis was employed and *p*<0.05 was considered statistically significant.

The potential confounding effects of BMI, length of breastfeeding, and time of introduction of cow’s milk on I-FABP level and on the level of IgA/IgG antibodies to beta-lactoglobulin, as well as to IgA/IgG antibodies to *Bifidobacterium* in the children’s cohort were analyzed by multiple regression analysis using the Jamovi statistical software version 2.6.26.

## 3 Results

### 3.1 The level of I-FABP and IgA/IgG to beta-lactoglobulin in the mothers and their offspring

The levels of I-FABP and IgA and IgG antibodies to bovine beta-lactoglobulin after delivery in the mothers, who had or had not a previous history of GDM, and in their children at TP1, are presented in **Tables 2**-**4**.

**Table 2:** The level of I-FABP and IgA and igG antibodies to beta-lactoglobulin in the mothers’ group.

Parameters	Total mothers’ group	Mothers with previous history of GDM	Mothers without GDM (Non-GDM)	P-value
I-FABP- level pg/ml				
n	40	19	21	
Median (25%-75%)	303.9 (124.8-674.8)	245.6 (112.2-490.0)	473.3 (147.0-791.4	P=0.31
beta-lactoglobulin IgA AU				
n	100	44	56	
Median (25%-75%)	0.10 (0.05-0.20)	0.10 (0.05-0.20)	0.11 (0.04-0.20)	P=0.34
beta-lactoglobulin IgG AU				
n	100	44	56	
Median (25%-75%)	0.30 (0.11-0.51)	0.2 (0.1-0.43)	0.37 (0.2-0.59)	p=0.0007

Mann-Whitney p values for the studied groups.

**Table 3:** The level of I-FABP and IgA and IgG antibodies to beta-lactoglobulin in the children’s group.

Parameters	Children at TP1	Children born to mothers who had GDM	Children born to non-GDM mothers	P-value
I-FABP- level pg/ml				
n	87	39	48	
Median (25%-75%)	476.5 (291.2-853.5)	435.8 (288.8-742.2)	522.8 (293.5-874.5)	P=0.82
beta-lactoglobulin IgA AU				
n	86	39	47	
Median (25%-75%)	0.16 (0.07-0.43)	0.3 (0.14-0.58)	0.12 (0.04-0.20)	P=0.004
beta-lactoglobulin IgG AU				
n	86	39	46	
Median (25%-75%)	0.70 (0.4-0.9)	0.70 (0.51-0.94)	(0.61 (0.39-0.90)	P=0.21

Mann-Whitney p values for the studied groups.

**Table 4:** The level of I-FABP and IgA and igG antibodies to beta-lactoglobulin in the mothers’ group vs childrens’ group.

Parameters	Total mothers’ group	Children at TP1	P-value
I-FABP- level pg/ml			
n	40	87	
Median (25%-75%)	303.9 (124.8-674.8)	476.5 (291.2-853.5)	P=0.009
beta-lactoglobulin IgA AU			
n	100	86	
Median (25%-75%)	0.10 (0.05-0.20)	0.16 (0.07-0.43)	P=0.007
beta-lactoglobulin IgG AU			
n	100	86	
Median (25%-75%)	0.30 (0.11-0.51)	0.70 (0.4-0.9)	P<0.0001

Mann-Whitney p values for the studied groups.

**Figure 1:**
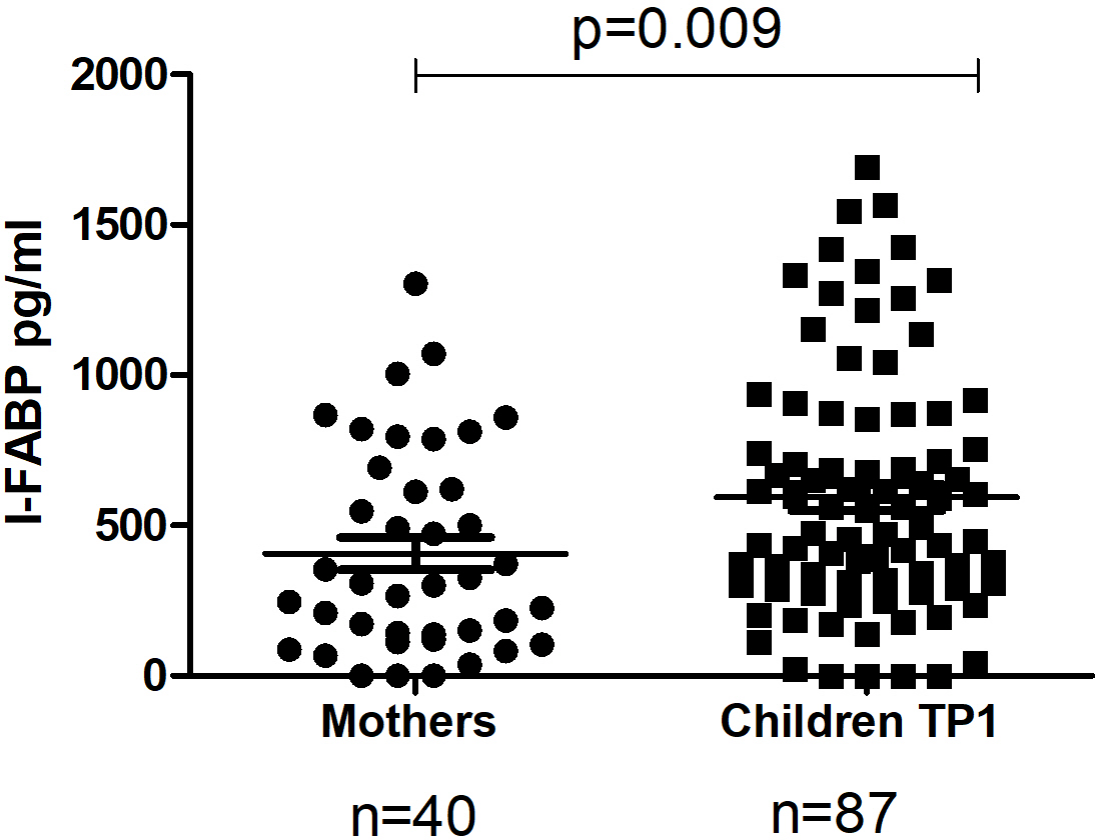
The level of I-FABP in the total mothers’ group and in their children at TP1. P-value calculated using the non-parametric Mann–Whitney test.

**Figure 2:**
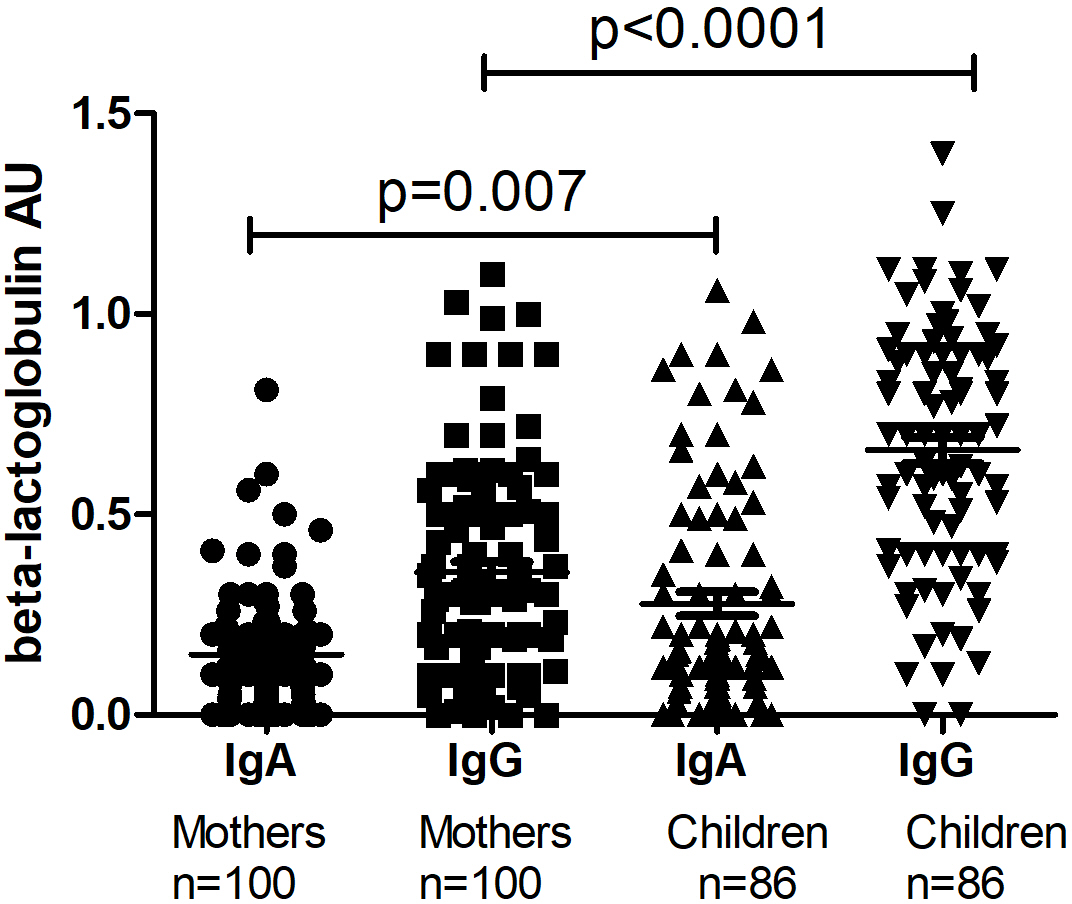
The level of IgA and IgG to beta-lactoglobulin in the total mothers’ group and in their children at TP1. P-value calculated using the non-parametric Mann–Whitney test.

The level of I-FABP was significantly higher in children at TP1 compared to the level of I-FABP for the total mothers’ group (p=0.009) (**Figure 1**). Also the level of IgA, as well as the level of IgG to beta-lactoglobulin were significantly higher for the children’s group compared to the mother’s group (p=0.007 and p<0.0001) (**Figure 2**).

The level of I-FABP, as well as the level of anti-beta-lactoglobulin IgA were significantly higher in children at TP1 compared to women with a previous history of GDM (p=0.005; p=0.0087) and the level of IgG to beta-lactoglobulin was significantly higher in children at TP1 compared to women with a previous history of GDM (p<0.0001) and non-GDM women (p<0.0001).

In the children’s group there occurred no significant difference in the level of I-FABP, as well as the level of IgA or IgG to beta-lactoglobulin between male and female children at TP1 (p=0.80; p=0.65 and p=0.62, respectively) (**Supplementary Table 1**).

The level of I-FABP, as well as the level of IgA to beta-lactoglobulin did not significantly differ between women with a previous history of GDM and non-GDM women (p=0.31 and p=0.34, respectively). However, the level of IgG to beta-lactoglobulin was significantly higher in non- GDM women compared to women with a previous history of GDM (p=0.0007).

The BMI of women with a previous history of GDM was significantly higher compared to non-GDM women (median BMI 27.6 *versus* 24.0; p= 0.04). However, there was no significant correlation between I-FABP level and BMI either in GDM (r= 0.06; p=0.80) or non-GDM (r=-0.35; p=0.14). Non-GDM mothers with BMI <25 showed a trend for higher I-FABP level (r=-0.35; p=0.14).

### 3.2 The level of I-FABP and IgA/IgG to beta-lactoglobulin in the children in relation to age

**Figure 3:**
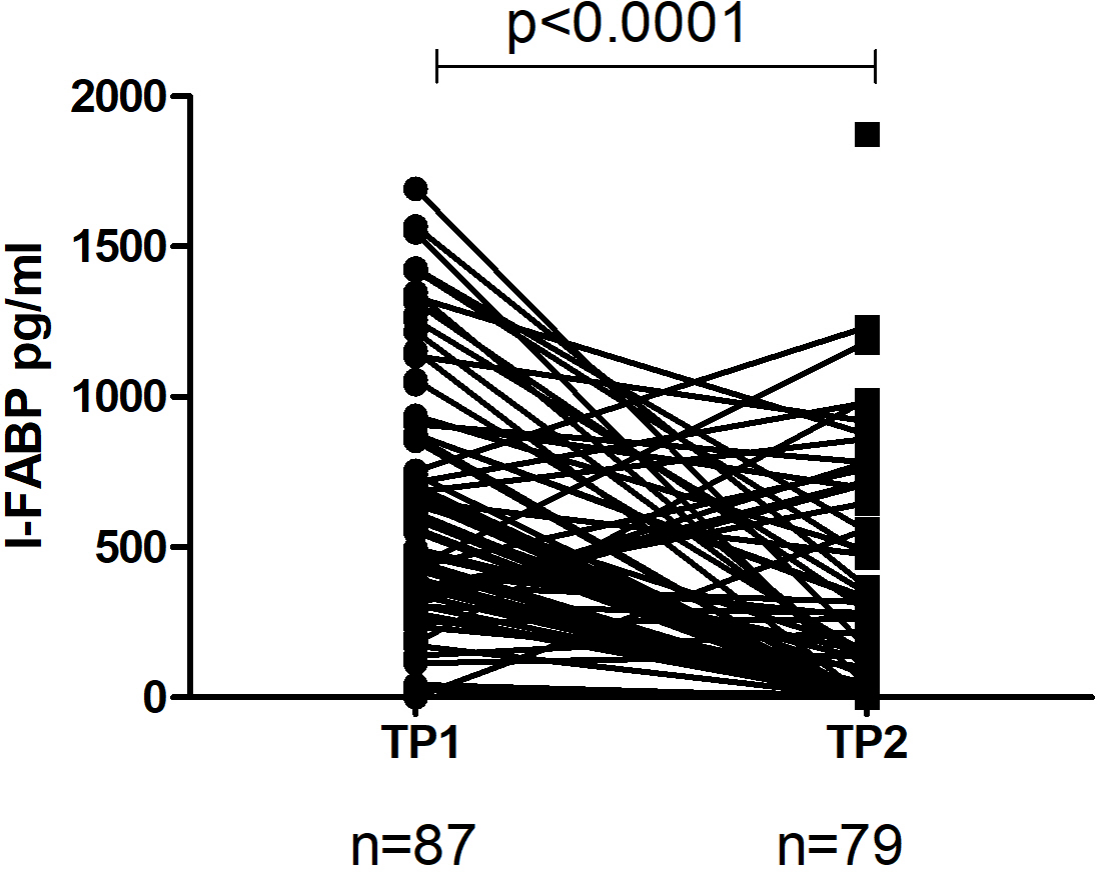
The level of I-FABP in children at TP1 and TP2. One line represents a change in I-FABP in child. P-value calculated using the Wilcoxon non-parametric rank test.

The level of I-FABP in the samples of children at TP1 was significantly higher (median 476.5 (291.2-853.5) compared to the level at TP2 (median 75.71 (0.0-468.83); p< 0.0001 (**Figure 3**).

**Figure 4:**
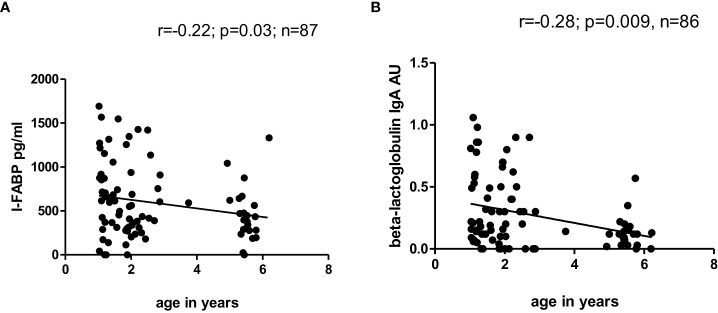
**(A, B)** Correlation between level of I-FABP and age and correlation between level of IgA to beta-lactoglobulin and age of children at TP1. P-value calculated using Spearman’s non-parametric rank correlation analysis.

Significant inverse correlation between level of I-FABP and age was observed for the total children’s group at TP1 (r=-0.22; p=0.03) (**Figure 4A**) and for male children (r=-0.37; p=0.007). Significant inverse correlation between level of IgA to beta-lactoglobulin and age was also found in the total children’s group at TP1 (r=-0.28; p=0.009) (**Figure 4B**).

### 3.3 The level of I-FABP and IgA/IgG to beta-lactoglobulin in the children at TP1 in relation to maternal GDM diagnosis

**Figure 5:**
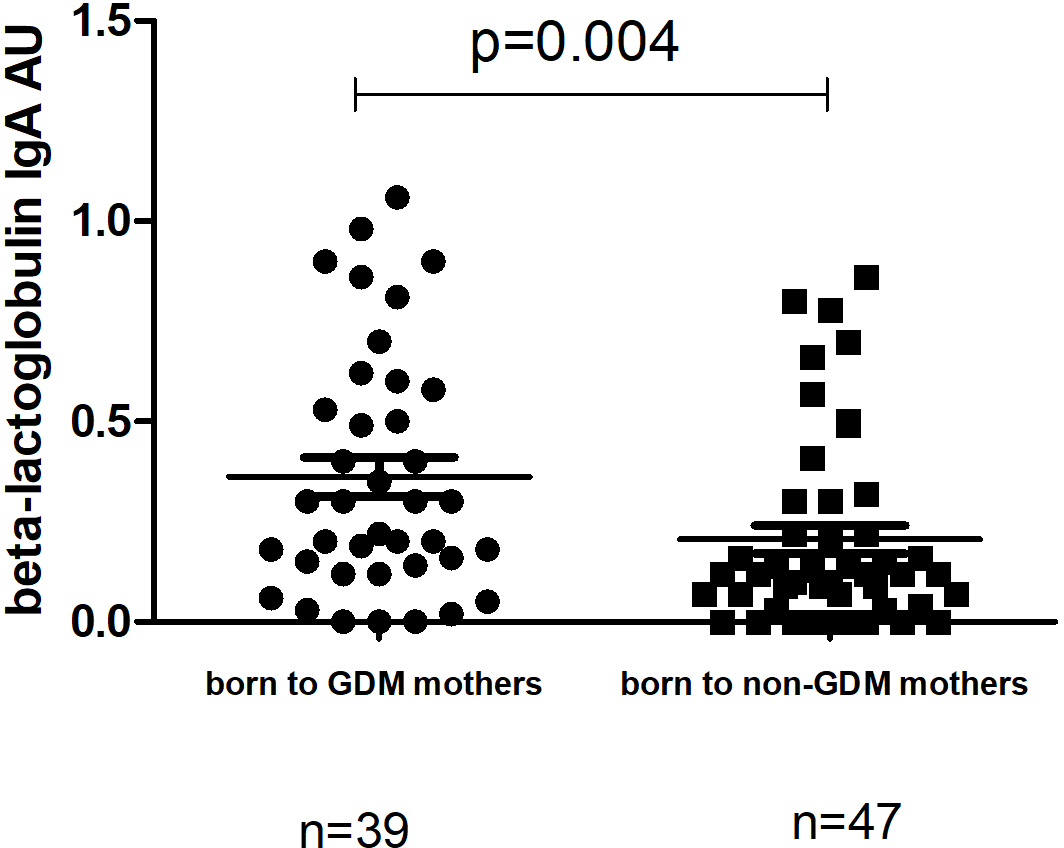
Comparison of the level of IgA to beta-lactoglobulin at TP1 between children at TP1 whose mothers had previous history of GDM (median 0.3 (0.14-0.58); mean±SE 0.36±0.04) and children born to non-GDM mothers (median 0.12 (0.04-0.30); mean ±SE 0.20±0.03). P-value calculated using non-parametric Mann–Whitney test.

The level of IgA to beta-lactoglobulin in children whose mothers had a previous history of GDM was significantly higher at TP1 (median 0.3 (0.14-0.58) compared to those whose mothers had no GDM (median 0.12 (0.04-0.30; p=0.004) (**Figure 5**) Neither the level of IgG to beta-lactoglobulin nor the level of I-FABP at TP1 differed significantly between children whose mothers had a previous history of GDM and those whose mothers were healthy (p=0.21 and p=0.82, respectively).

### 3.4 The level of IgG4 to the beta-lactoglobulin component f77 nBos d5 in the children at TP1 and TP2

The level of IgG4 to beta-lactoglobulin in the samples of children did not differ significantly at TP1and TP2 (median level 2.07 (0.21-17.95) vs 3.29 (0.52-17.4); p=0.35).

**Figure 6:**
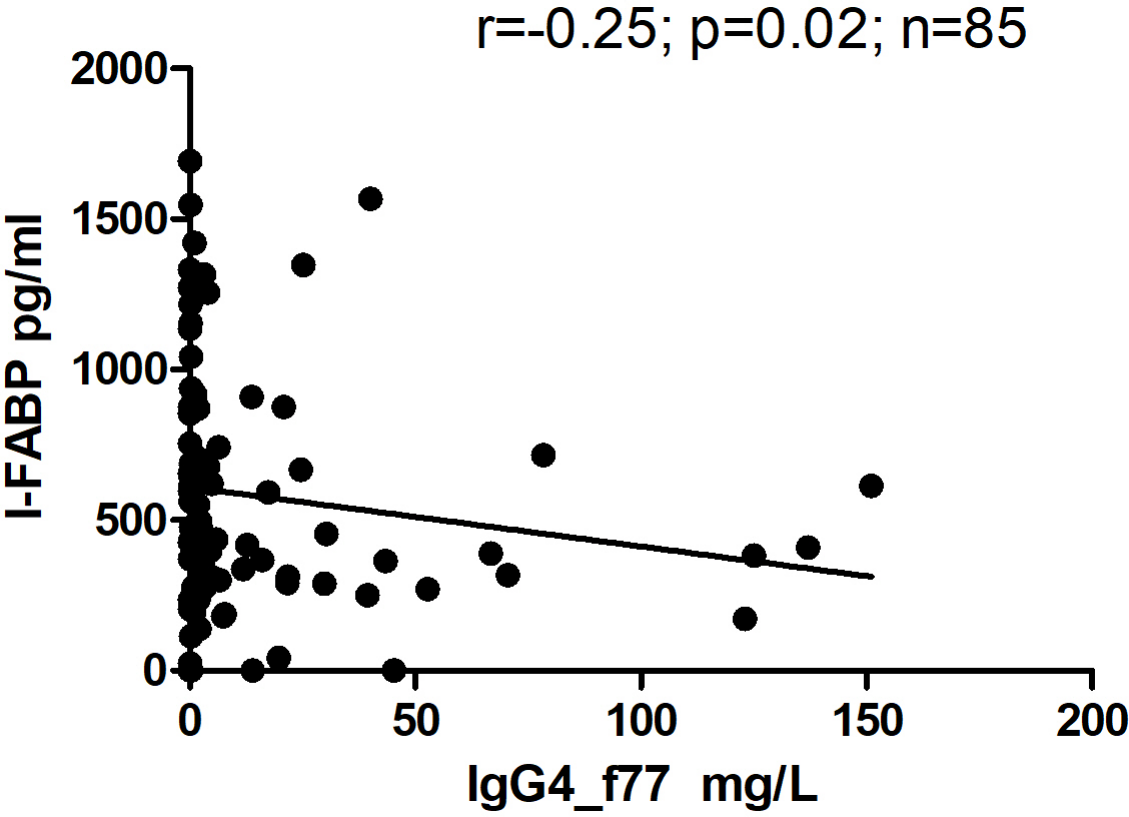
Correlation between level of I-FABP and level of IgG4 to the beta-lactoglobulin component nBosd5 in children at TP1. P-value calculated using Spearman’s non-parametric rank correlation analysis.

**Figure 7:**
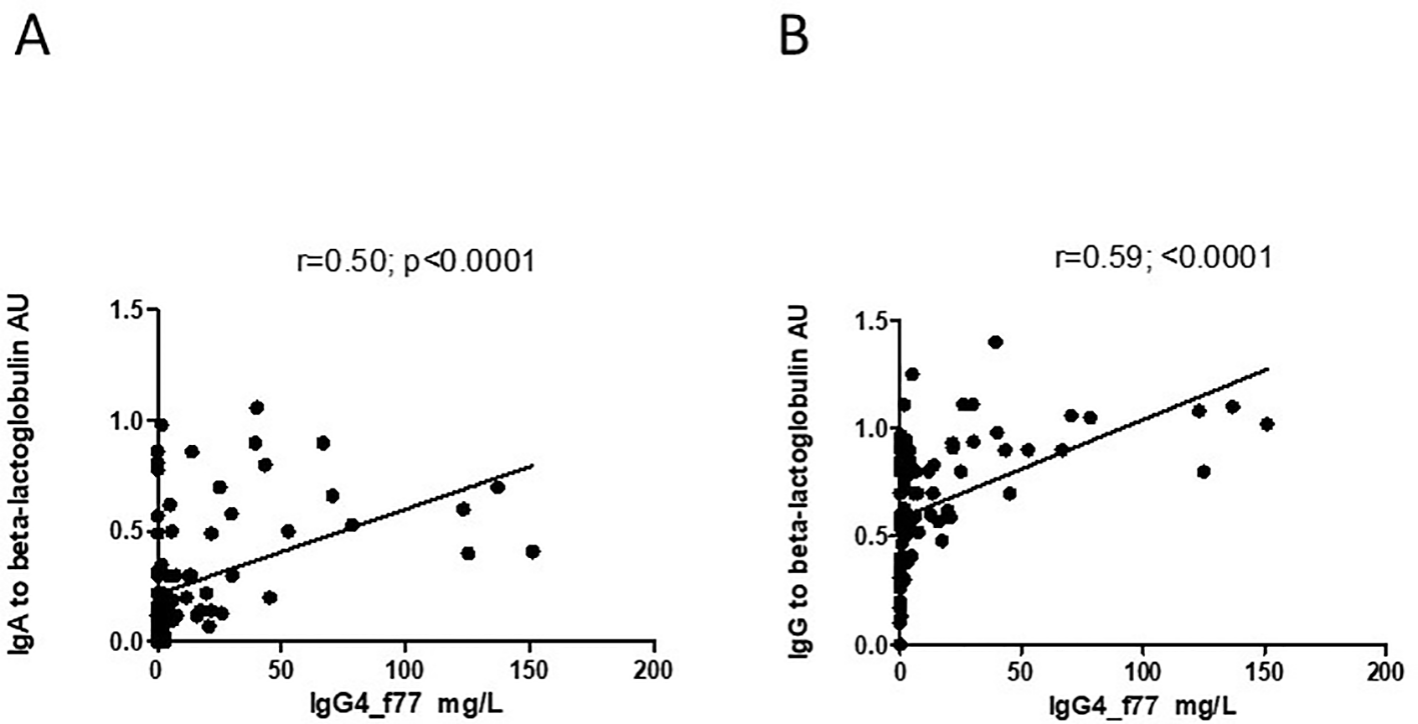
**(A, B)** Correlation between level of IgG4 to the beta-lactoglobulin component nBosd5 and level of IgA and IgG to beta-lactoglobulin in children at TP1. P-value calculated using Spearman’s non-parametric rank correlation analysis.

However, there was significant inverse correlation between level of IgG4 and level of I-FABP for children sampled at TP1 (r=-0.25; p=0.02) (**Figure 6**). Level of IgG4 to beta-lactoglobulin correlated positively with level of IgG, as well as with level of IgA to beta-lactoglobulin for children sampled at TP1(r=0.59; p<0.0001 and r=0.50; p<0.0001) (**Figures 7A, B**).

### 3.5 The level of I-FABP and IgA/IgG antibodies to *Bifidobacterium* in the mothers with a previous history of GDM, non-GDM mothers and their children at TP1

**Figure 8:**
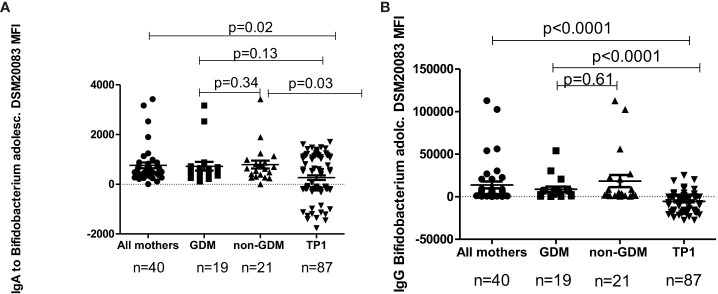
**(A)** The level of IgA to *B. adolescentis* strain DSM20083 in all mothers, in GDM mothers, in non-GDM mothers and in children at TP1. P-value calculated using the non-parametric Mann–Whitney test. **(B)** The level of IgG to *B. adolescentis* strain DSM20083 in all mothers, in GDM mothers, in non-GDM mothers and in children at TP1. P-value calculated using the non-parametric Mann–Whitney test.

**Figure 9:**
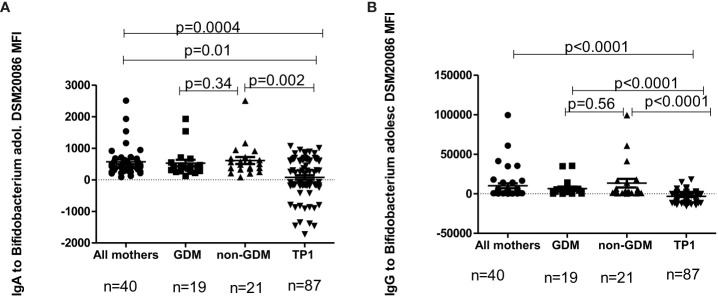
**(A)** The level of IgA to *B.adolescentis* strain DSM20086 in GDM mothers, in non-GDM mothers and in children at TP1. P-value calculated using the non-parametric Mann–Whitney test. **(B)** The level of IgG to *B. adolescentis strain* DSM20086 in GDM mothers, in non-GDM mothers and in children at TP1. P-value calculated using the non-parametric Mann–Whitney test.

The level of IgA/IgG antibodies to *B.adolescentis* strains DSM20083 and DSM20086 for the total mothers’ group, in GDM mothers, in non-GDM mothers and in the samples of children at TP1, evaluated by flow cytometry, is presented in **Figures 8A, B**, **9A, B**.

In children the level of IgA to *B.adolescentis* (DSM20083) at TP1 was significantly lower compared to the total mothers’ group (p=0.02), as well as to non-GDM women (p=0.03), but not compared to women with a previous history of GDM (p=0.13). At the same time, the level of IgG to *B. adolescentis* (DSM20083) at TP1 was significantly lower in children compared to the total mothers’ group (p<0.0001), women with GDM (p<0.0001) and non-GDM women (p<0.0001).

In children the level of IgA to *B. adolescentis* (DSM20086) at TP1 was also significantly lower compared to the total mothers’ group (p=0.0004), women with a previous history of GDM (p=0.01) and non-GDM women (p=0.002). The level of IgG to *B. adolescentis* (DSM20086) in the samples at TP1 was significantly lower compared to the total mothers’ group (p<0.0001), GDM women (p<0.0001) and non-GDM women (p<0.0001).

The data about the correlation between level of I-FABP and IgA/IgG to beta-lactoglobulin and level of IgA/IgG antibodies to *B. adolescentis* strains DSM20083 and DSM20086 for the total mothers’ group, and for women with or without a previous history of GDM is presented in **Table 5**.

**Table 5:** Correlation of levels of I-FABP, IgA/IgG to beta-lactoglobulin with level of IgA/IgG antibodies to *B.* DSM20083 and DSM20086 strains for the total mothers’ group, for mothers with previous history of GDM and for non-GDM mothers.

Parameters	I-FABP pg/ml			beta-lactoglobulin IgA AU			beta-lactoglobulin IgG AU		
	Total mothers’ group	GDM	Non- GDM	Total mothers’ group	GDM	Non- GDM	Total mothers’ group	GDM	Non- GDM
Level of IgA to *B.adolescentis* DSM20083									
n	40	19	21	40	19	21	40	19	21
r	-0.34	-0.68	-0.08	0.15	-0.13	0.30	0.12	0.03	0.29
p	0.03	0.0013	0.71	0.35	0.57	0.17	0.44	0.88	0.18
Level of IgG to *B.adolescentis* DSM20083									
n	40	19	21	40	19	21	40	19	21
r	-0.05	-0.14	-0.04	0.09	0.24	0.07	0.12	0.37	-0.11
p	0.74	0.55	0.86	0.57	0.31	0.73	0.44	0.11	0.62
Level of IgA to *B.adolescentis* DSM20086									
n	40	19	21	40	19	21	40	19	21
r	-0.25	-0.72	0.12	0.17	-0.07	0.33	0.17	0.07	0.36
p	0.11	0.0005	0.59	0.27	0.77	0.14	0.27	0.75	0.09
Level of IgG to *B.adolescentis* DSM20086									
n	40	19	21	40	19	21	40	19	21
r	-0.03	-0.20	0.04	0.09	0.16	0.09	0.16	0.40	-0.06
p	0.82	0.40	0.84	0.55	0.49	0.68	0.30	0.08	0.79

**Figure 10:**
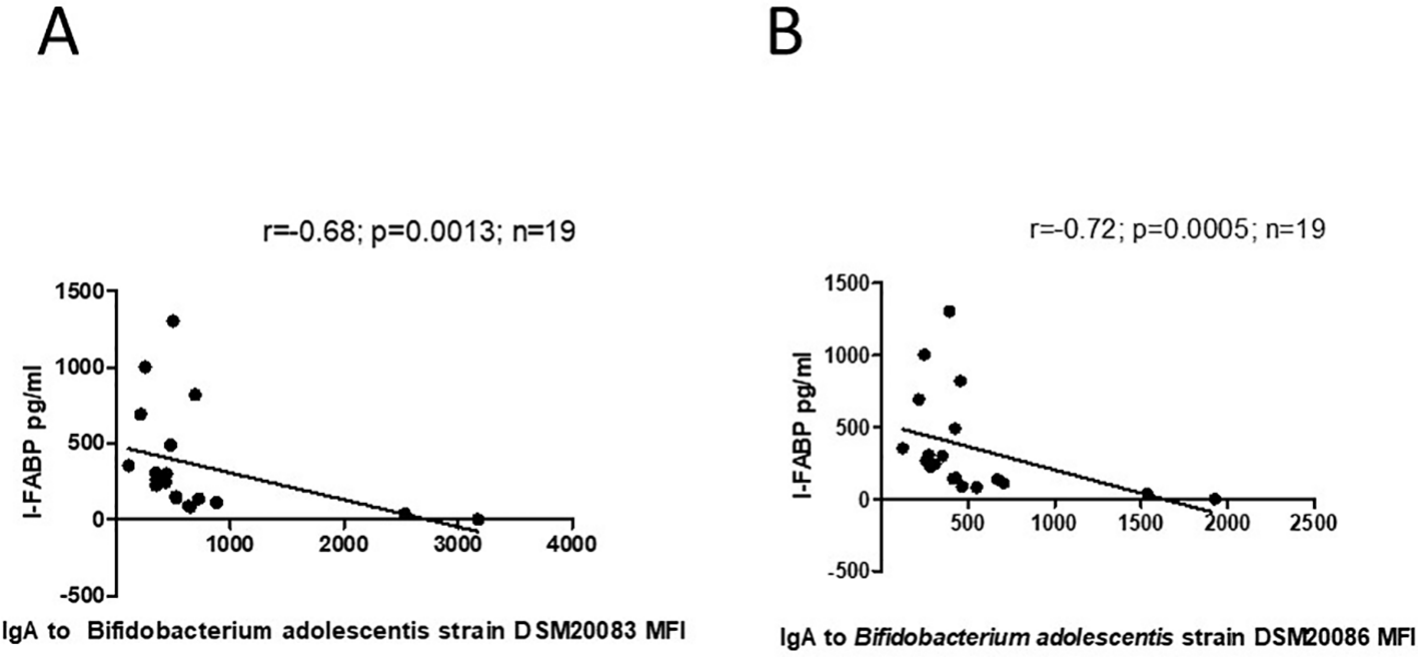
**(A)** Correlation between level of I-FABP and IgA to *B.adolescentis* strain DSM20083 in mothers with a previous history of GDM. P-value calculated using Spearman’s non-parametric rank correlation analysis. **(B)** Correlation between level of I-FABP and IgA to *B. adolscentis* strain DSM20086 in mothers with a previous history of GDM. P-value calculated using Spearman’s non-parametric rank correlation analysis.

For mothers with a previous history of GDM, significant inverse correlation was found between level of I-FABP and level of IgA to *B. adolescentis* strain DSM20083 (r=-0.68; p=0.0013), as well as to *B.adolescentis* strain DSM20086 (r=-0.72; p=0.0005). (**Figures 10A, B**).

However, there was no significant correlation between level of beta-lactoglobulin IgA or IgG and level of IgA or IgG antibodies to *B.adolescentis* (strain DSM20083 or DSM20086) for the group of GDM women (**Table 5**).

For children there was no significant correlation between level of I-FABP and level of IgA or IgG to the *B.adolescentis* strain DSM20083 at TP1 (r=0.15; p=0.14 and r=-0.11; p=0.28 respectively), as well as between level of I-FABP and level of IgA or IgG_to the *B. adolescentis* strain DSM20086 (r= 0.16; p=0.12 and r=-0.13; p=0.21, respectively). No significant correlation occurred between level of IgA or IgG to beta-lactoglobulin and antibodies to *B.adolescentis* strain DSM20083 and DSM20086. There was a mere trend for correlation between level of IgA to beta-lactoglobulin and IgA to *B. adolescentis* strain DSM20083 for children at TP1 (r=0.21; p=0.05) (**Supplementary Table 2**).

### 3.6 Correlation between level of I-FABP, IgA/IgG to bovin beta-lactoglobulin and number of IgA/IgG bands to *Bifidobacterium breve* strain DSM20213 for children at TP1

No significant correlation was found between level of I-FABP and number of IgA/IgG bands to *B. breve* strain DSM20213 (r=-0.11, p=0.32; r=-0.04, p=0.71) for children at TP1. There was a weak trend for negative correlation between level of IgA to beta-lactoglobulin and number of IgA bands *to B. breve* strain DSM20213 (r=-0.18; p=0.09), as well as and between level of IgG to beta-lactoglobulin and number of IgA bands to *B. breve* strain DSM20213 (r=-0.21; p=0.05).

However, significant positive correlation was found between number of IgA bands to *B. breve* strain DSM20213 and age of children at TP1 (r=0.46; p<0.0001) (**Supplementary Figure 1**).

### 3.7 The level of I-FABP and the level of IgA and IgG to beta-lactoglobulin in the children at TP1 in relation to atopic dermatitis/asthma or atopic rhinitis diagnosis

There was no significant difference in the level of I-FABP or the level of IgA and IgG to beta-lactoglobulin between children with atopic dermatitis or asthma diagnosis and healthy children (p>0.05) (**Supplementary Tables 3**, **4**).

### 3.8 The level of I-FABP and the level of IgA and IgG to beta-lactoglobulin in the children at TP1 and TP2 in relation to the length of breastfeeding

There was no significant difference in the level of I-FABP between children who received breastfeeding and those who did not; nor did this level depend on the length of breastfeeding for the groups of children at TP1 and TP2 (p>0.01). The level of IgA to beta-lactoglobulin at TP1 was significantly higher in children who received breast milk until 6 months compared to those who did not receive breast milk (p=0.02) and compared to those who received breast milk longer than 12 months (p=0.0003). The level of IgG beta-lactoglobulin was significantly higher in children who received breast milk until 6 months compared to those who did not receive breast milk (p=0.02) or received it until 7–12 months (p=0.04) and compared to those who received breast milk longer than 12 months (p=0.0003) (**Supplementary Tables 5**, **6**).

### 3.9 The level of IgA and IgG antibodies to beta-lactoglobulin and the level of I-FABP in the children at TP1 in relation to the time of introduction of cow’s milk

The median time when children sampled at TP1 received cow’s milk was 12.0 months (min. 6.0 and max 20.0 months.). There was no significant difference in the level of IgA or IgG antibodies to beta-lactoglobulin between children who received cow’s milk for the first time earlier than 12 months after birth and those who received it later (**Supplementary Figures 2A, B**). Nor did the level of I-FABP correlate with the time of introduction of cow’s milk (r=-0.06; p=0.53).

### 3.10 Association of the level of beta-lactoglobulin IgA/IgG antibodies and the level of I-FABP with postnatal hypoglycemia

The level of IgA and IgG antibodies to beta-lactoglobulin and the level of I-FABP were compared in children with postnatal hypoglycemia (diagnosed according to locally used guidelines on monitoring neonatal hypoglycemia used in Estonia), in children who required oral formula feeding, and in children without hypoglycemia (**Supplementary Table 7** and **Supplementary Figures 3**, **4A, B**). There was found no significant difference either in the level of I-FABP or the level of IgA/IgG to beta-lactoglobulin between these groups of children (p>0.05).

### 3.11 Linear regression model

Using multiple regression analysis for evaluation of the contribution of the age of children, length of breastfeeding, month of introduction of cow’s milk, BMI and diagnosis of mothers to the level of I-FABP and IgA/IgG antibodies to beta-lactoglobulin, we found significant negative association of I-FABP level with children’s age (β=-66.4; p=0.01) (adR^2^ = 0.04) irrespective of length of breastfeeding (β=8.97; p=0.23), month of introduction of cow’s milk (β=3.76; p=0.8), BMI (β=3.44; p=0.55) or diagnosis of mothers (β=39.41; p=0.66).

The level of IgA to beta-lactoglobulin was inversely associated with length of breastfeeding, (β=-0.014; p=0.004), age of children (β=-0.04; p= 0.007) (adR^2^ = 0.20) and diagnosis of mothers (β=0.14, p=0.009). The level of IgA to beta-lactoglobulin was not dependent on the month of introduction of cow’s milk (β=0.008; p=0.37).

The level of IgG to beta-lactoglobulin was also inversely associated with length of breastfeeding (β=0.011; p=0.03) (adR^2^ = 0.07) and was not dependent on the month of introduction of cow’s milk (β= -0.01; p=0.34).

Using a linear regression model for evaluation of the effect of diagnosis of mothers, age of children, length of breastfeeding and month of introduction of cow’s milk on the level of IgA/IgG antibodies to *Bifidobacterium* in the children’s cohort we found that the level of IgA/IgG antibodies to the *Bifidobacterium* in the children cohort was not dependent on diagnosis of mothers, length of breastfeeding or month of introduction of cow’s milk (p<0.05).

## 4 Discussion

The essential finding of our study was that the level of I-FABP, as well as the level of IgA to beta-lactoglobulin did not differ significantly between women with a previous history of GDM and non-GDM controls. However, the level of IgG to beta-lactoglobulin was significantly higher in healthy non-GDM persons.

The BMI of mothers with a previous history of GDM was significantly higher compared to BMI in the non-GDM group. However, there was no significant correlation between the level of I-FABP and BMI either for the GDM or for the non-GDM group. Non-GDM healthy mothers showed a trend for higher level of I-FABP for those with BMI <25, which is consistent with our previous results (9).

The level of I-FABP, as well as the level of IgA and IgG to bovine beta-lactoglobulin were significantly higher in children (TP1) compared to the mothers’ group.

Significant inverse correlation between level of I-FABP and age was observed for the total group of children at TP1 and for male children. Also, significant inverse correlation was found between level of IgA to beta-lactoglobulin and age for the total group of children and a trend for it for male children. With increasing age the level of I-FABP, as well as the level of IgA to beta-lactoglobulin decreased significantly in the studied children. These results could be explained by the fact that children’s gut permeability is higher compared to adults (28). According to literature data, intestinal permeability to macromolecules is very high shortly after birth and drops within the first week. Intestinal permeability decreases faster in breast-fed infants. After weaning from breast milk, gut permeability temporarily increases again (28, 29). Kalach et al. (2001) (30) also showed that intestinal permeability correlates negatively with age in control children and is altered in children with cow’s milk allergy. Breastfeeding plays an important role in gut permeability and contributes to gut “closure”, because human milk, especially colostrum, contains several gut trophic factors like epidermal growth factors (29, 31). However, in our study there was no significant difference in I-FABP level between children who received breastfeeding and those who were not breastfed; nor did this level depend on the length of breastfeeding either at TP1 or at TP2 (p>0.01).

The level of I-FABP did not differ significantly at TP1 between children whose mothers had a previous history of GDM and those whose mothers were healthy. Only the level of IgA to beta-lactoglobulin was significantly higher in children whose mothers had GDM during pregnancy compared to those whose mothers did not have GDM. According to some authors, the level of IgA and IgG to beta-lactoglobulin in pediatric patients with T1D was significantly higher than that in the control group (Kohno et al., 2002) (32). Increase of IgA to beta-lactoglobulin in offspring in our study can be attributed to maternal GDM during pregnancy. A study of Luopajärvi et al. (2008) (12) investigating children with T1D showed that children who developed T1D had enhanced immune reactivity to dietary cow’s milk proteins already in infancy. According to these authors, infants may exhibit stronger immune reactions to oral antigens due to either increased intestinal permeability or a lag in the maturation of their gut immune system. Increased gut permeability can result from gut dysbiosis, which is characteristic of mothers with GDM and can be transferred to their offspring (33, 34). In addition, low-grade systemic inflammation is a significant feature of GDM and has been observed in both affected women and their offspring (35). Dysbiosis-induced gut permeability associated with elevated inflammation can impact the development of the child’s immune system, potentially priming the fetal immune system for elevated reactivity to dietary antigens (36, 37). In line with this, maternal GDM has been associated with increased sensitization to food allergens in offspring during early childhood (38).

Vaarala et al. (1995) (39) reported that the oral introduction of cow’s milk proteins in healthy children in early infancy induced development of IgG antibodies to beta-lactoglobulin, bovine serum albumin and alfa- casein, as well as T cell response to these components. However, later, T-cell response declined, which supports the concept of oral tolerance. In our study, there was no significant difference in the level of IgA or IgG antibodies to beta-lactoglobulin at TP1 between children who received cow’s milk for the first time before 12 months after birth and those who received it for the first time later than 12 months after birth.

### 4.1 I-FABP and immune response to *B.adolescentis* and to *B. breve*

For mothers, there was significant inverse correlation between level of I-FABP and IgA to the *B. adolescentis* strain DSM20083 between the total group and those with a previous history of GDM. Higher level of I-FABP was accompanied by lower level of IgA to *B. adolscentis* strain DSM20083 as well as to *B.adolescentis strain* DSM20086 in the GDM group. There are data about alterations in the maternal microbiome during pregnancy, especially in women with GDM (40–42). It has been reported that changes in the gut microbiome can lead to increased intestinal permeability and translocation of bacterial components in the blood stream, which can lead to insulin resistance via inflammatory response (20, 40, 41). A decrease in beneficial bacteria, e.g. *Bifidobacterium*, was shown in women with GDM (41, 43). Importantly, commensal gut microbes can induce the response of serum IgA and IgG to facilitate their colonization and functioning (44). *B.adolescentis* is an important component of the microbiome that may preserve intestinal barrier (19).

There was no significant correlation between level of I-FABP and level of IgA or IgG to *B.adolescentis* strain DSM20083, as well as between level of I-FABP and level of IgA or IgG to *B.adolescentis strain* DSM20086 for children at TP1. Only a weak trend for correlation was noted between level of IgA to beta-lactoglobulin and IgA to *B. adolescentis* strain DSM20083, as well as between level of I-FABP and level of IgA to *B.breve* strain DSM20213 at TP1.

No significant correlation occurred between level of I-FABP and number of IgA/IgG immunoblot bands to *B. breve* strain DSM20213 at TP1.

Significant positive correlation was found between number of IgA bands to *B.breve* and age. The response of IgA to B. breve increased with the age of children.

The role of the increased abundance of *B.breve* and the importance of breastfeeding in the maturation of intestinal barrier has been highlighted in the literature (45).

*B. breve* is a known as a dominant *Bifidobacterium* species in the gut microbiota of both preterm and term infants (46), while *B.breve* has most commonly been recovered from among bifidobacterial species isolated from the breast milk of healthy women (47).

However, in the present study we did not reveal a significant difference in I-FABP level, which might reflect the state of intestinal barrier, between children who received breastfeeding and those who did not. The level of I-FABP was not dependent on the length of breastfeeding for the children’s group either at TP1 or TP2 (p>0.01).

The level of IgA/IgG to beta-lactoglobulin was significantly higher in children who received breastfeeding until 6 months after birth compared to those who did not receive it, as well as compared to those who received breastfeeding longer than 12 months. Ma et al. (2022) (45) have stressed the critical role of sufficient breast milk feeding and the specific capability of *Bifidobacterium* for carbohydrate metabolism in coordinated postnatal intestinal barrier improvement.

Several literature data point to the diversity of the microbiota in children with allergic disease (48, 49). According to Kalliomäki M et al. (2001) (49), differences in the gut microflora precede the development of atopy, while children suffering from allergies have smaller amounts of *Bifidobacterium* species and larger amounts of Clostridium bacteria compared with healthy children. This diversity of the microbiota might influence the gut permeability in children with atopic disease.

However, our study established no significant difference in the level of I-FABP or in the level of IgA and IgG to beta-lactoglobulin between children with the diagnosis of atopic dermatitis or asthma and healthy children.

Our study has several limitations, such as the relatively small number of the persons studied in the mothers’ group. On the other hand, the strength of the study is the circumstance that their offspring were investigated at two time points. The mothers and their children were invited to visit the study pediatrician twice, with a one- year intent between each visit, which allowed to assess gut permeability and immune response beta-lactoglobulin in dynamics.

The study highlights the possible consequence of age in the association between the commensal microbiota and development of immune mediated disorders in the offspring of mothers with higher risk of GDM. We would like to emphasize the fact that the permeability of the intestinal mucosa in children changes during growth and depends on child’s age which should be taking into account in the clinical management of children.

## 5 Conclusions

The level of I-FABP and the level of antibodies to beta-lactoglobulin were not significantly different between mothers with a previous history of GDM and healthy mothers.

The level of both I-FABP and the level of IgA to beta-lactoglobulin significantly decreased with age in the studied children and was lower in the mothers, which indicates that intestinal permeability is higher in children compared to adults.

Only the level of IgA (but not IgG) to beta-lactoglobulin was significantly higher in the children whose mothers had a previous history of GDM compared to the children whose mothers did not have GDM.

In the GDM group, there was significant inverse correlation between the level of I-FABP and the level of IgA against both strains of *B.adolescentis*. Number of *B.breve* IgA bands correlated with age of the children.

Atopic dermatitis or asthma in children does not affect the level I-FABP or the levels of IgA/IgG to beta-lactoglobulin.

There was no significant difference in the level of I-FABP between the children who received breastfeeding and those who did not received it, nor was it dependent on the length of breastfeeding in the children at TP1 or TP2.

Levels of IgA and IgG to beta-lactoglobulin was inversely associated with length of breastfeeding and were not dependent on the month of introduction of cow’s milk.

## Data Availability

The datasets presented in this article are not readily available due to confidentiality restrictions. Requests to access the datasets should be directed to the corresponding author TV.

## Supplementary material

Supplementary Figure 1 Correlation between age of children at TP1 and number of bands of IgA to *B.breve strain DSM20213.* P-value calculated using Spearman’s non-parametric rank correlation analysis.

Supplementary Figure 2
**(A, B)**. The level of IgA and IgG to beta-lactoglobulin in children TP1 who received cow’s milk before 12 months of birth and in children who received it later than after 12 months after birth. P-value calculated using non-parametric Mann–Whitney test.

Supplementary Figure 3 Comparison of the level of I-FABP at TP1 in children who received formula feeding after birth and in children who did not received formula feeding after birth. P-value calculated using non-parametric Mann–Whitney test.
