# Supplementary figures and images for: The level of I-FABP and IgA/IgG to beta-lactoglobulin in mothers at risk for gestational diabetes and in their children: association with antibodies to Bifidobacterium adolescentis and Bifidobacterium breve
Source: Front Immunol. 2025 Jul 2;16:1613002. doi: 10.3389/fimmu.2025.1613002 (PMC12263606; doi:10.3389/fimmu.2025.1613002)

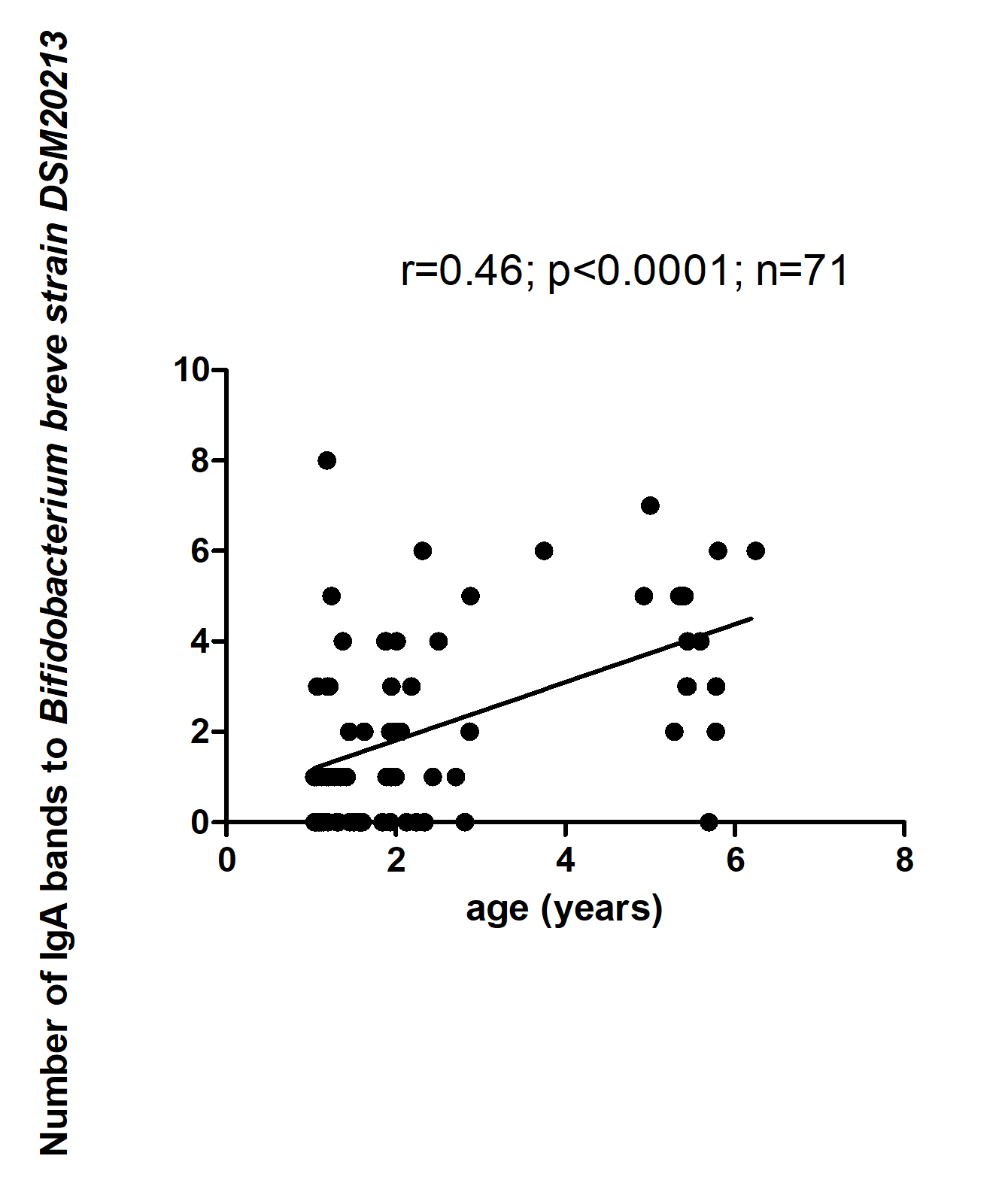

Supplement: Supplementary Figure 1 — Correlation between age of children at TP1 and number of bands of IgA to B.breve strain DSM20213. P-value calculated using Spearman’s non-parametric rank correlation analysis. [file Image1.jpeg]

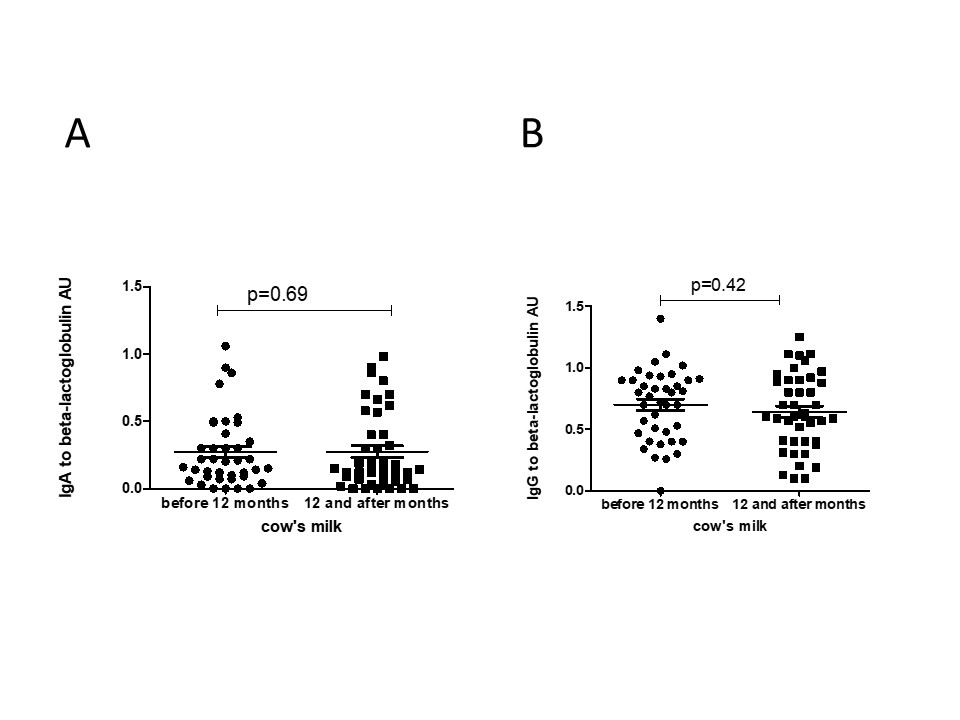

Supplement: Supplementary Figure 2 — (A, B). The level of IgA and IgG to beta-lactoglobulin in children TP1 who received cow’s milk before 12 months of birth and in children who received it later than after 12 months after birth. P-value calculated using non-parametric Mann–Whitney test. [file Image2.jpeg]

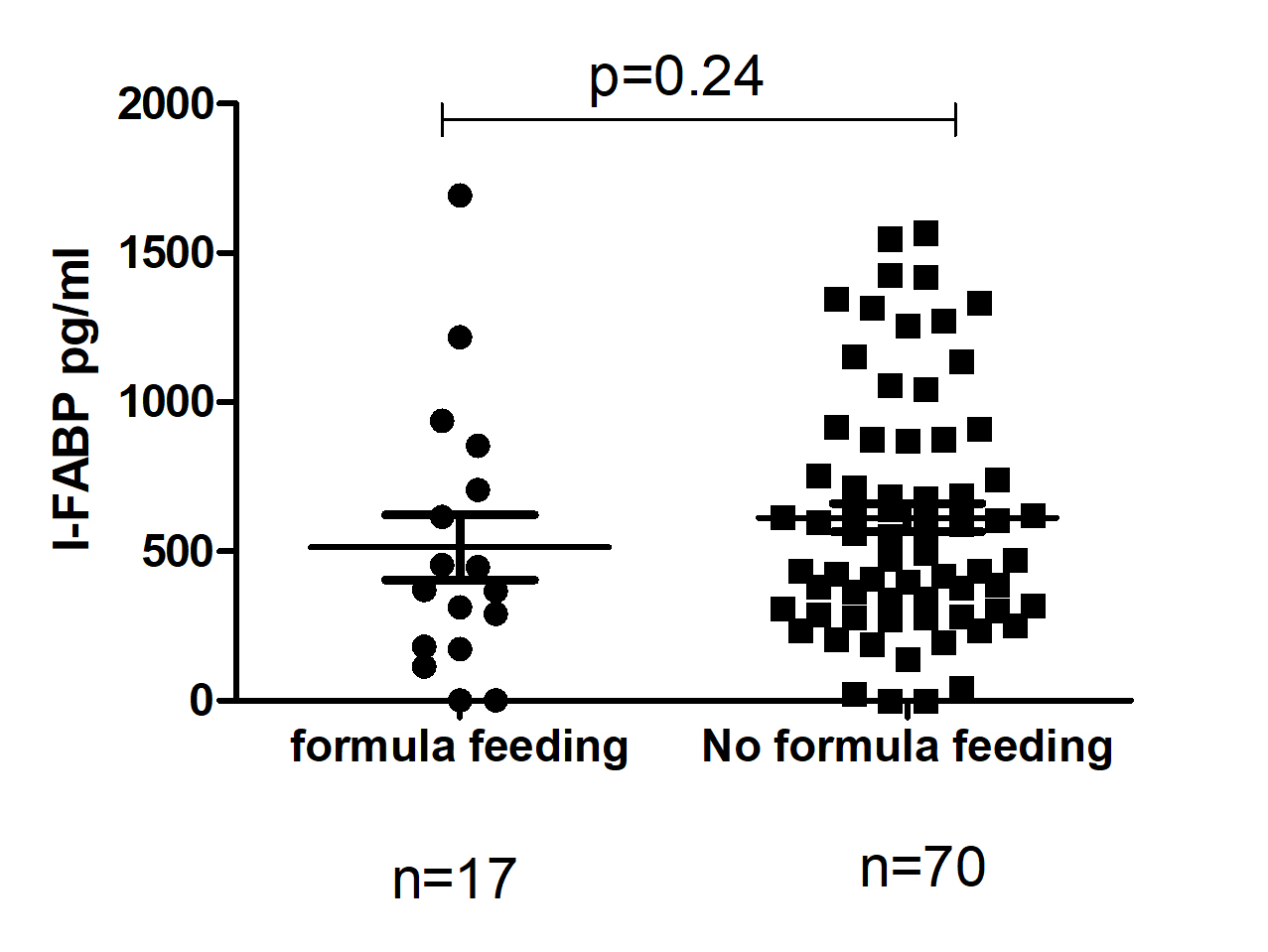

Supplement: Supplementary Figure 3 — Comparison of the level of I-FABP at TP1 in children who received formula feeding after birth and in children who did not received formula feeding after birth. P-value calculated using non-parametric Mann–Whitney test. [file Image3.jpeg]

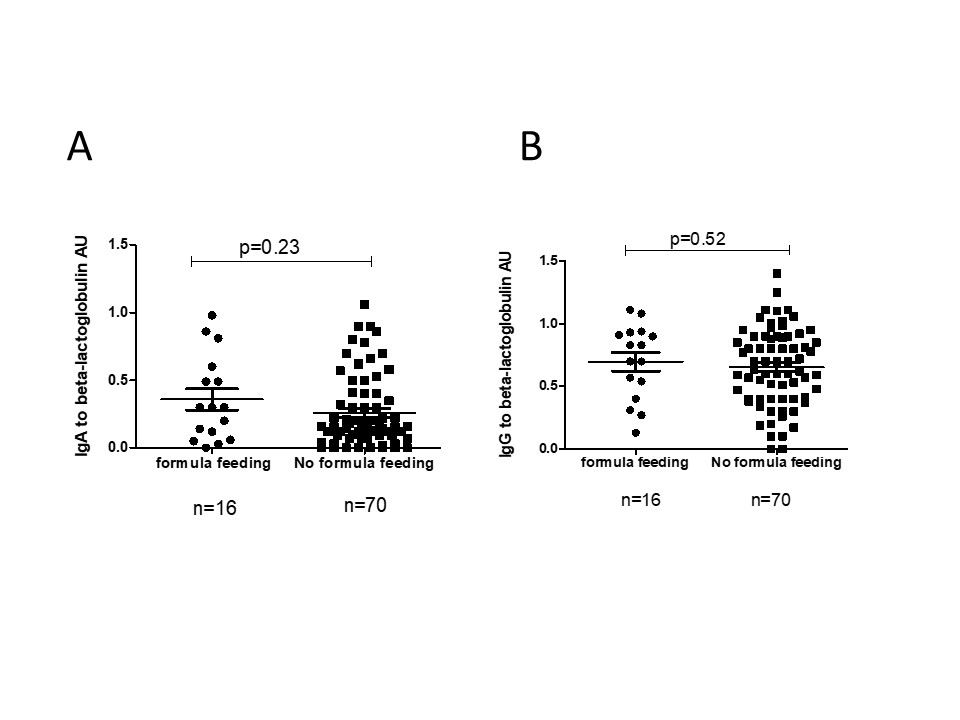

Supplement: Supplementary file 4 [file Image4.jpeg]
