# Supplementary material for: The level of I-FABP and IgA/IgG to beta-lactoglobulin in mothers at risk for gestational diabetes and in their children: association with antibodies to Bifidobacterium adolescentis and Bifidobacterium breve
Source: Front Immunol. 2025 Jul 2;16:1613002. doi: 10.3389/fimmu.2025.1613002 (PMC12263606; doi:10.3389/fimmu.2025.1613002)
Supplement: Supplementary file 5 [file Table1.pdf]

Supplementary Table. S1. The level of I-FABP and IgA and IgG antibodies to beta-lactoglobulin in children at TP1 (male and female).

	**I-FABP- level pg/ml**		**beta-lactoglobulin IgA AU**		**beta-lactoglobulin IgG AU**	
	Children at TP1		Children at TP1		Children at TP1	
	male	female	male	female	male	female
n	49	38	48	38	48	38
Median (25%-75%)	495.0 (310.3-797.9)	473.6 (275.3-922.1)	0.15 (0.07-0.49)	0.20 (0.06-0.40)	0.66 (0.4-0.88)	0.74 (0.4-0.9)
Mann-Whitney		P=0.83		P=0.65		P=0.62

Supplementary Table S2. Correlation of level of I-FABP, level of IgA/IgG to beta-lactoglobulin with level of IgA /IgG to *B.adolscentis* strains DSM20083, DSM20086 and *B.breve* strain DSM20213 for children at TP1.

		I-FABP pg/ml	beta-lactoglobulin IgA AU	beta-lactoglobulin IgG AU
		Children at TP1	Children at TP1	Children at TP1
IgA_ to *B. adolescentis* DSM20083				
	n	85	84	84
	r	0.15	0.21	0.18
	p	0.14	**0.05**	0.09
IgG to *B. adolescentis* DSM20083				

	n	85	84	84
	r	-0.11	-0.12	-0.03
	p	0.28	0.26	0.77
IgA to *B. adolescentis* DSM20086				
	n	85	84	84
	r	0.16	0.18	0.15
	p	0.12	0.09	0.15
IgG to *B. adolescentis* DSM20086				
	n	85	84	84
	r	-0.13	-0.15	-0.04
	p	0.21	0.15	0.68
IgA to *B. breve* DSM20213				
	n	86	85	85
	r	0.08	0.17	0.06
	p	0.43	0.10	0.55
IgG to *B.breve* DSM20213				
	n	86	85	85
	r	-0.19	-0.10	-0.08
	p	0.06	0.33	0.44
Number of IgA bands to B. breve DSM20213				

	n	81	81	81
	r	-0.11	-0.18	-0.21
	p	0.32	0.09	0.05
Number of IgG bands to B. breve DSM20213				
	n	81	81	81
	r	-0.04	-0.04	0.05
	p	0.71	0.69	0.60

Supplementary Table S3. Comparison of the level of I-FABP in children with atopic dermatitis and asthma/allergic rhinitis and in healthy children at TP1.

	**I-FABP- level pg/ml**			
	Children at TP1 with atopic dermatitis	Healthy children at TP1	Children at TP1 with asthma/allergic rhinitis	Healthy Children at TP1
n	27	60	13	74
Median (25%-75%)	370.0 (203.5-853.5)	562.0 (338.1-841.7)	397.1 (279.7-516.2)	577.3 (298.2-875.8)
Mann-Whitney		0.24		0.19

Supplementary Table S4. Comparison of the level of IgA and IgG to beta-lactoglobulin in children with atopic dermatitis and asthma/allergic rhinitis and in healthy children at TP1.

	**beta-lactoglobulin IgA AU**				**beta-lactoglobulin IgG AU**			
	Children at TP1 with atopic dermatitis	Healthy children at TP1	Children with asthma/allergic rhinitis at TP1	Healthy Children at TP1	Children with atopic dermatitis at TP1	Healthy children at TP1	Children with asthma/allergic rhinitis at TP1	Healthy Children at TP1
n	25	61	13	73	25	61	13	73
Median (25%-75%)	0.16 (0.07-0.4)	0.16 (0.07-0.45)	0.12 (0.03-0.39)	0.16 (0.07-0.45)	0.63 (0.4-0.88)	0.7 (0.4-0.91)	0.53 (0.3-0.93)	0.7 (0.4-0.9)
Mann-Whitney		0.84		0.46		0.51		0.51

Supplementary Table S5. The level of I-FABP in children at TP1 and TP2 in relation to the length of breastfeeding

	**I-FABP- level pg/ml in children at TP1**				**I-FABP- level pg/ml in children at TP2**			
	Length of breastfeeding				Length of breastfeeding			
months	0	until 6	7-12	>12	0	until 6	7-12	>12
n	2	17	16	52	3	18	15	44
Median (25%-75%)	783.9 (235.3-1333)	388.8 (285.0-606.1)	609.0 (348.7-700.6)	528.4 (293.5-901.0)	102.9 (0.0-875.7)	17.86 (0.0-663.2)	47.14 (0.0-468.6)	86.43 (0.0-343.6)

Mann-Whitney p in I-FABP at TP1: 0 and until 6 months p=0.94; until 6 and 7-12 months p=0.24; until 6 months and > 12 months p=0.26; 7-12 and > 12 months p=0.90; 0 and >12 months p=0.76.

Mann-Whitney p in I-FABP at TP2: 0 and 6 months p=0.79; 6 and 7-12 months p=0.89; 6 and >12 months p=0.87; 0 and <12 months p=0.75.

Supplementary Table S6. The level of IgA/IgG to beta-lactoglobulin in children at TP1 in relation to the length of breastfeeding

	**IgA to beta-lactoglobulin level AU in children at TP1**				**IgG to beta-lactoglobulin level AU in children at TP1**			
	Length of breastfeeding				Length of breastfeeding			
months	0	until 6	7-12	>12	0	until 6	7-12	>12
n	2	17	14	51	2	17	15	51
Median (25%-75%)	0.01 (0.0-0.03)	0.53 (0.09-0.8)	0.36 (0.1-0.55)	0.14 (0.05-0.22)	0.15 (0.0-0.3)	0.90 (0.56-1.05)	0.80 (0.57-1.02)	0.70 (0.37-0.83)

Mann-Whitney p: Beta-lactoglobulin IgA : 0 and until 6 months p=0.02; until 6 and >12 months p=0.0003

Mann-Whitney p: Beta-lactoglobulin IgG : 0 and until 6 months p=0.02; until 6 and >12 months p=0.003; 0 and 7-12 p=0.04; 0 and > 12 months p=0.05

Supplementary Table S7. The level of I-FABP and IgA/IgG to beta-lactoglobulin in children with postnatal hypoglycemia who received formula feeding and in children without postnatal hypoglycemia who did not receive formula feeding at TP1.

	**I-FABP- level pg/ml in children at TP1**		**IgA to beta-lactoglobulin level AU In children at TP1**		**IgG to beta-lactoglobulin level AU in children at TP1**	
	with postnatal hypoglycemia	Without postnatal hypoglycemia,	with postnatal hypoglycemia	Without postnatal hypoglycemia,	with postnatal hypoglycemia	Without postnatal hypoglycemia,
n	17	70	16	70	16	70
Median (25%-75%)	370.0 (176.4-779.9)	556.2 8306.8-872.2)	0.30 (0.07-0.57)	0.15 (0.07-0.40)	0.76 (0.43-0.92)	0.66 (0.40-0.90)
Mann-Whitney		P=0.24		P=0.23		P=0.52
